# SARS-CoV-2 spike protein induces salivary gland dysfunction and immune infiltration in C57BL/6 mice

**DOI:** 10.3389/fimmu.2025.1667597

**Published:** 2025-11-21

**Authors:** Fernanda Aragão Felix, Yuqiao Jiang, Jing Zhou, Dongfang Li, Victoria Zhou, Sílvia Ferreira de Sousa, Kevin Matthew Byrd, Qing Yu

**Affiliations:** 1 The ADA Forsyth Institute, Somerville, MA, United States; 2 Department of Oral Surgery, Pathology and Clinical Dentistry, School of Dentistry, Universidade Federal de Minas Gerais, Belo Horizonte, Minas Gerais, Brazil; 3 Lab of Oral & Craniofacial Innovation, ADA Science & Research Institute, Gaithersburg, MD, United States; 4 Department of Oral and Craniofacial Molecular Biology, Philips Institute for Oral Health Research, Virginia Commonwealth University, Richmond, VA, United States

**Keywords:** SARS-CoV-2 virus, COVID-19, spike protein, salivary gland dysfunction, Sjögren’s disease, Sjögren’s syndrome

## Abstract

Salivary gland dysfunction and inflammation are common following SARS-CoV-2 infection. This study aimed to investigate the effects of SARS-CoV-2 spike and envelope proteins on glandular function in healthy C57BL/6 mice through direct intra-glandular injection into the submandibular glands. Local administration of spike protein significantly reduced salivary secretion, while the envelope protein had no measurable impact. Histological analysis revealed the presence of leukocyte foci in two-thirds of spike protein-treated mice, while none were detected in the vehicle- or envelope protein-treated groups. Furthermore, spike protein treatment led to a significant increase in total immune cells and B cells, and an expansion of the CD44^high^ CD62L^low^ effector/effector memory subsets within CD4 T cells and B cells in the submandibular glands. Notably, serum antinuclear antibodies developed in one-third of spike-treated mice, consistent with the reported salivary gland pathology in COVID-19 patients that resemble autoimmune Sjögren’s disease. Moreover, spike protein treatment increased phospho-STAT3 levels and induced transcriptomic changes indicating impaired acinar compartment, heightened adaptive immune responses, and altered tissue remodeling activity. These findings show that SARS-CoV-2 spike protein alone is sufficient to initiate significant salivary gland pathology in the absence of intact virus or ACE2 interaction, providing evidence for a novel mechanism by which SARS-CoV-2 induces salivary gland dysfunction and pathology with autoimmune features.

## 1 Introduction

Normal salivary production is critical for maintaining oral and systemic health (1). Salivary gland function and integrity can be perturbed by microbial infections, autoimmune diseases, aging and tumors (1, 2). Salivary glands are vulnerable to viral infections and saliva serves as an important medium for the transmission of viruses (1, 3). Viral infections are common triggers and contributors to salivary gland dysfunction and inflammatory pathology (1, 2). Some examples include Epstein–Barr virus (EBV), cytomegalovirus (CMV) (3, 4), hepatitis C virus (HCV) (3), and severe acute respiratory syndrome coronavirus 2 (SARS-CoV-2) (5). Notably, various manifestations of viral infections of the salivary glands, such as reduced salivary secretion and immune infiltration of the glands (6–8), resemble the characteristics of Sjögren’s disease (SjD), a chronic autoimmune condition primarily affecting the salivary and lacrimal glands but also causing systemic manifestations (4).

Since its emergence in late 2019, the SARS-CoV-2 virus, a single-stranded RNA virus of the Coronaviridae family, has caused widespread illness and significant mortality globally (9–11). Beyond the initial respiratory symptoms, the virus is associated with diverse oral and systemic complications (12), including persistent symptoms collectively known as ‘Long COVID’ (13). SARS-CoV-2 RNA and its spike (S) protein, a key structural component, have been detected in salivary glands and saliva of patients following SARS-CoV-2 infection (5, 14, 15). Increasing evidence points to the virus’s impact on the salivary glands, resulting in xerostomia (dry mouth), glandular enlargement, and leukocyte infiltration in COVID-19 and Long COVID patients (8, 12, 16–18). The direct impact of SARS-CoV-2 virus is further confirmed with transgenic mice expressing the human ACE2 receptor, showing that SARS-CoV-2 infection impairs saliva production while inducing lymphocyte infiltration of salivary and lacrimal glands and the emergence of anti-SSA/SSB autoantibodies (8). These changes in mice mirror those observed in COVID-19 patients (8).

SARS-CoV-2 enters cells via S protein binding to ACE2, triggering anti-viral and inflammatory responses through viral RNA interaction with intracellular sensors like retinoic acid-inducible gene I (RIG-I), melanoma differentiation-associated protein 5 (MDA5), and Toll-like receptor (TLR) 3 and -7 (19–22). However, additional mechanisms may also contribute to SARS-CoV-2’s effects on salivary glands. Studies have shown that cell surface TLR2 and TLR4 recognize SARS-CoV-2 S protein or envelope (E) protein to induce hyperinflammatory responses in lung and brain tissues, suggesting that these proteins can provoke inflammatory pathology independently of viral entry and replication (23–25). Importantly, salivary gland epithelial cells express TLR2 and TLR4 in healthy human subjects, with increased expression in SjD patients (19, 26, 27). Hence, SARS-CoV-2 structural proteins may contribute to chronic salivary gland dysfunction reported in many post-COVID patients (17, 18). To evaluate this hypothesis, we exposed salivary glands of wild-type C57BL/6 mice to recombinant SARS-CoV-2 S and E proteins, and found that S protein alone is sufficient to induce notable salivary gland pathology resembling that seen in COVID-19 patients.

## 2 Methods

### 2.1 Animals

C57BL/6J (C57BL/6) mice were purchased from the Jackson Laboratory and housed in the specific pathogen-free animal facility at the ADA Forsyth Institute. All protocols were approved by the Forsyth IACUC and complied to the “Guide for the Care and Use of Laboratory Animals” of the National Institutes of Health and the ARRIVE guidelines.

### 2.2 *In Vivo* administration of recombinant S and E protein

Both recombinant S protein and E protein (RP01283LQ and RP01263LQ) (24, 28) were purchased from Abclonal as sterilized solutions, with >95% purity and endotoxin activity < 0.1 EU/μg of the protein. 10-week-old female C57BL/6 mice were anesthetized with a mixture of oxygen and isoflurane gas. Initially oxygen was given at 1.5 L/min and the isoflurane at 5% and subsequently reduced to 1% for maintenance. After mice lost consciousness, 50 µl of PBS or PBS solution containing 1 µg S protein or E protein was directly injected into each of the two submandibular gland (SMG) lobes. Mice were monitored until they regain consciousness and display normal behavior. The injection was performed every three days over a two-week period. The salivary flow rate was measured two days after the final injection. The following day, mice were euthanized by CO_2_ inhalation (displacement rate at 30–70% of the chamber volume/min) in accordance with AVMA Guidelines for the Euthanasia of Animals. CO2 flow was maintained for 1 min after respiratory arrest. After death was confirmed, blood and tissues were collected from the mice.

### 2.3 Measurement of salivary flow rate

Mice were weighed and intraperitoneally injected with 100 μl PBS-based secretagogue solution containing isoproterenol (1 mg/ml) and pilocarpine (2 mg/ml) as we described (29–31). One min later, saliva was collected continuously for 5 min with a micropipette. Saliva volume was measured and normalized to the body weight.

### 2.4 Antibody staining and flow cytometry

Single cells were prepared from freshly harvested submandibular glands (SMGs) or submandibular gland lymph nodes (SMLNs), incubated with anti-CD16/32 (clone 93) before being stained with a combination of fluorescence-conjugated antibodies (BioLegend) to specific surface markers, including CD45 (clone 30-F11), CD4 (clone GK1.5), CD8α (clone 53-6.7), CD19 (clone 6D5), EpCAM (clone G8.8), CD62L (clone MEL-14), and CD44 (clone IM7). After washing, the stained cells were analyzed using an Attune NxT Flow Cytometer (Invitrogen) and subsequently the FlowJo V10 software.

### 2.5 Immunohistochemistry and assessment of leukocyte infiltration of SMGs

Formalin-fixed, paraffin-embedded SMGs were sectioned and stained with hematoxylin and eosin (H&E). The number of leukocyte foci (a cluster/aggregate of cells containing at least 50 leukocytes), and leukocyte focus score (number of leukocyte foci within a 4 mm^2^ tissue area) were determined. For immunohistochemistry, SMG sections were subjected to antigen retrieval, blockade of endogenous peroxidase activity, and inhibition of non-specific binding with 5% goat serum. The sections were incubated overnight with the following primary antibodies: anti-TNFα (clone ab6671, Abcam), anti-phosphorylated NFκB p65 (clone PA5-118567, Invitrogen), anti-phosphorylated STAT3 (clone 13A3-1, BioLegend), or anti-CXCL10 (clone A16079E, BioLegend). After further incubation with secondary antibodies, immunodetection was performed using VECTASTAIN Elite ABC Kits (Vector Laboratories) followed by 3,3’-diaminobenzidine for color development and hematoxylin for counterstaining. The sections were imaged and the brown signals were quantified in Image J 1.50i using a semiautomatic threshold method as we previously described (32–34). Briefly, red, blue, and green color thresholding was done to achieve appropriate segmentation of the brown stained areas. The percentage of brown areas was measured in 8–10 fields per SMG section.

### 2.6 Antinuclear antibody detection by enzyme-linked immunosorbent assays

Serum ANA levels were determined using Mouse ANA ELISA kit (Biomatik) following the manufacturer’s instructions. HRP-streptavidin conjugate and TMB substrate were used for the color development, and the absorbance at 450 nm and 570 nm was measured on a microplate reader (BioTek). The adjusted optical density (OD450-570) was calculated by subtracting the absorbance at 570 nm (reference wavelength) from that at 450 nm. Mice with adjusted OD > 0 were considered ANA-positive.

### 2.7 RNA-sequencing and bioinformatics

RNA-sequencing was performed by Azenta Life Sciences. Briefly, cDNA libraries were generated from RNA isolated from SMG cells with poly-A selection and sequenced on the Illumina NovaSeq X Plus platform at the depth of 20–30 million reads per sample. All samples were processed and sequenced in a single batch. Raw sequencing data (.bcl files) were converted to fastq files and de-multiplexed using Illumina’s bcl2fastq 2.17. The qualified sequencing reads were aligned to mouse genome reference sequence (UCSC mm10, NCBI). The unique gene hit counts were determined using the Feature Counts tool (Subread v.1.5.2). Differentially expressed genes (DEGs) between S protein and PBS-treated groups were identified using the DESeq2 R package. P-values were adjusted using the Benjamini-Hochberg correction, and DEGs with adjusted p-values < 0.05 and absolute log2 fold change > 1 were identified as significant DEGs. Kyoto Encyclopedia of Genes and Genomes (KEGG) pathway analysis, REACTOME pathway analysis, and Gene Ontology (GO) function and enrichment analysis were performed on the significant DEGs using Database for Annotation, Visualization, and Integrated Discover (DAVID) (35, 36). Gene Set Enrichment Analysis (GSEA) was performed on all DEGs using the software developed by the Broad Institute (5, 35) and the Hallmark mouse gene set collection from the Molecular Signatures Database (MSigDB; mouse h.all.v25.1.hs.symbols.gmt, v25.1). In all pathway analyses, pathways with a false discovery rate (FDR) < 0.05 were considered as significantly enriched.

### 2.8 Statistical analysis

Statistical analyses were performed using the Graph Pad Prism software. Two-tailed Student’s t-test or Mann-Whitney U test was performed to assess differences between two groups as appropriate. P values smaller than 0.05 were considered statistically significant.

## 3 Results

### 3.1 Local exposure of salivary gland tissues to SARS-CoV-2 S induces glandular pathology and autoantibody production in C57BL/6 mice

We used only female mice in this study because SjD exhibits a striking female predominance, which is largely recapitulated in most murine models (20, 37–42). While SARS-CoV-2-induced salivary secretory dysfunction affects both sexes at similar rate in humans and mice (16, 17, 43, 44), female patients and mice infected by this virus show higher levels of serum ANA compared to the males, and female mice infected with the virus also display greater salivary tissue apoptosis (8). We therefore used female mice only in this work. Recombinant S (S1+S2) protein, E protein, or control PBS was transcutaneously injected into both SMG lobes of 10-week-old female C57BL/6 mice as we previously described (33), every three days over a two-week period. The dosage was selected within the range used for injections to mice in published reports (23, 45) and the injection regimen was designed to mimic the sustained antigen presence that occurs during acute SARS-CoV-2 infection.

**Figure 1:**
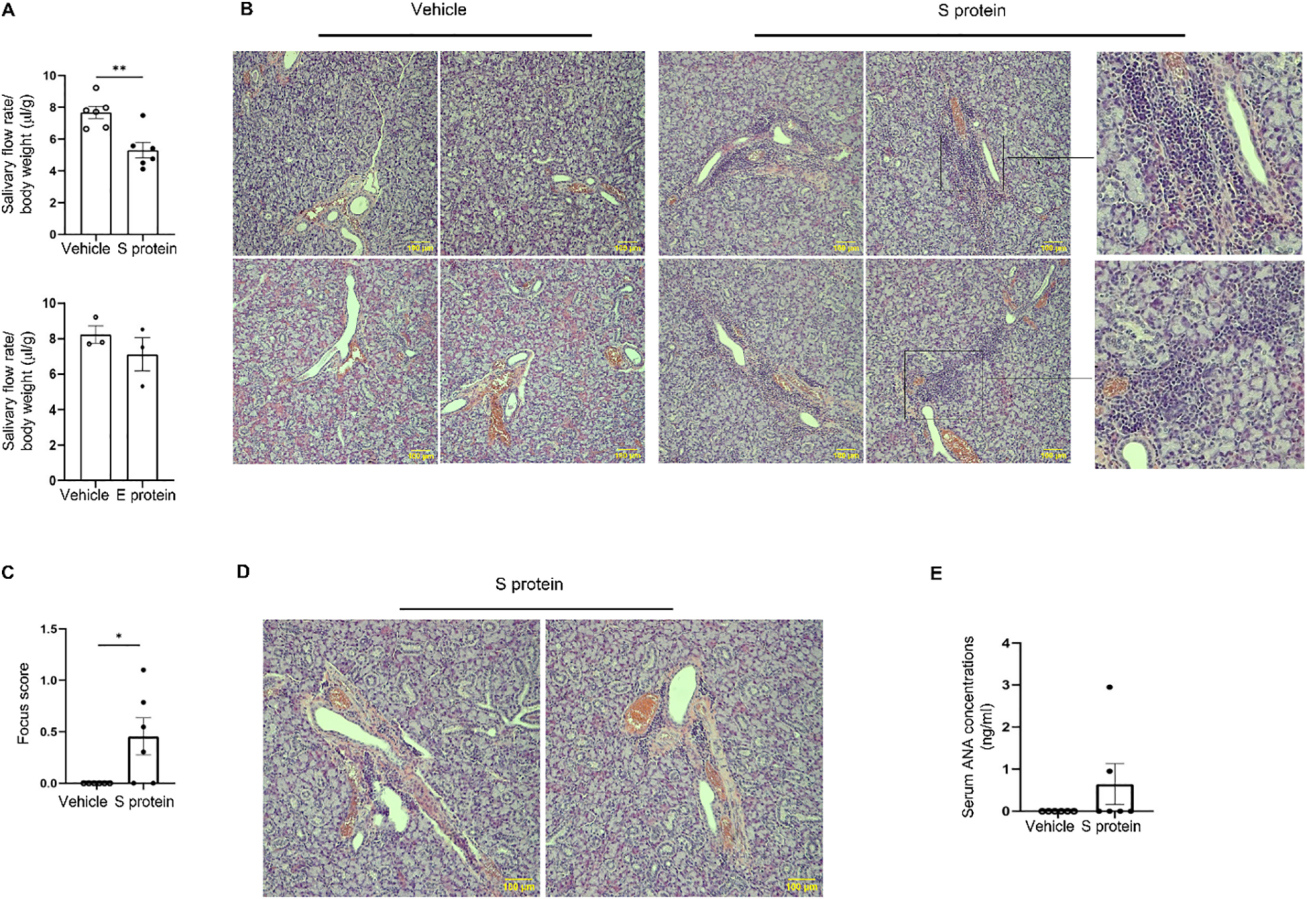
Exposure of salivary gland tissues to recombinant SARS-CoV-2 S protein induces SjD-like salivary gland pathology in C57BL/6 mice. Recombinant S (S1+S2) protein, E protein, or the control PBS solution was directly injected into both lobes of the submandibular glands (SMGs) of 10-week-old female C57BL/6 mice at 1 µg/lobe every three days for a total of 4 times. The salivary flow rate was measured, and tissues were harvested 2–3 days after the final injection. **(A)** Salivary flow rate normalized to body weight (n=6 mice/group). Left: Vehicle control versus S protein treatment. Right: Vehicle control versus E protein treatment. Values are the mean ± standard error of the mean (SEM), which is the case for all other graphs in this paper. **(B)** Representative images of SMG sections stained with hematoxylin and eosin (×200 magnification) containing clear leukocyte foci, with magnified insets (on the right) showing selected regions from some of the images. Scale bars, 100 μm. **(C)** The graph shows the leukocytic focus score, defined as the number of leukocytic foci within a 4 mm^2^ tissue area (n=6 mice each group). **(D)** Representative images of hematoxylin & eosin-stained SMGs (×200 magnification) containing small leukocyte infiltrates in the S protein-treated group (n=6 mice each group). **(E)** Relative levels of antinuclear antibody (ANA) in 1:10 diluted sera as determined by ELISA (n=6 mice each group). Data are expressed as mean ± standard error of mean (SEM). Statistical significance was determined using two-tailed Student’s t-test or Mann-Whitney U test as appropriate, with P values < 0.05 considered as significant. *P < 0.05, **P < 0.01.

Intra-SMG administration of S protein, but not E protein, caused a significant reduction in the salivary flow rate (**Figure 1A**). Histological analysis of SMGs revealed the presence of leukocyte foci in approximately two-thirds of S protein-treated mice, primarily located around ducts and blood vessels (**Figure 1B**). By contrast, no leukocyte foci were observed in the PBS-treated or E protein-treated group (**Figure 1B**). Accordingly, the leukocyte focus score was significantly higher in the S protein-treated group (**Figure 1C**). Moreover, although the classically defined leukocyte foci were absent in one-third of the S protein-treated mice, smaller immune cell aggregates were observed, primarily around blood vessels, and to a lesser extent, near ducts (**Figure 1D**). By contrast, these small immune aggregates were not observed in the control group. Therefore, both leukocyte foci and the smaller immune aggregates were induced by S protein treatment but not the control treatment.

It has been shown that some COVID-19 patients display positive serum ANA and anti-SSA (46, 47). In accordance, serum ANA assay showed that two out of six (33.3%) of the S protein-treated mice developed ANA autoantibodies (**Figure 1E**), closely mirroring the reported prevalence of ANA positivity (20-30%) in COVID-19 patients (8, 47). This finding suggests that S protein may play a role in the development of autoimmune disorders, such as SjD, in a subset of COVID-19 patients. In summary, exposure of SMGs of healthy C57BL/6 mice to S protein alone is sufficient to trigger notable pathology resembling SjD seen in COVID-19 patients.

### 3.2 Exposure of salivary gland tissues to SARS-CoV-2 S protein leads to an increase in the activated T- and B cells within the glands

**Figure 2:**
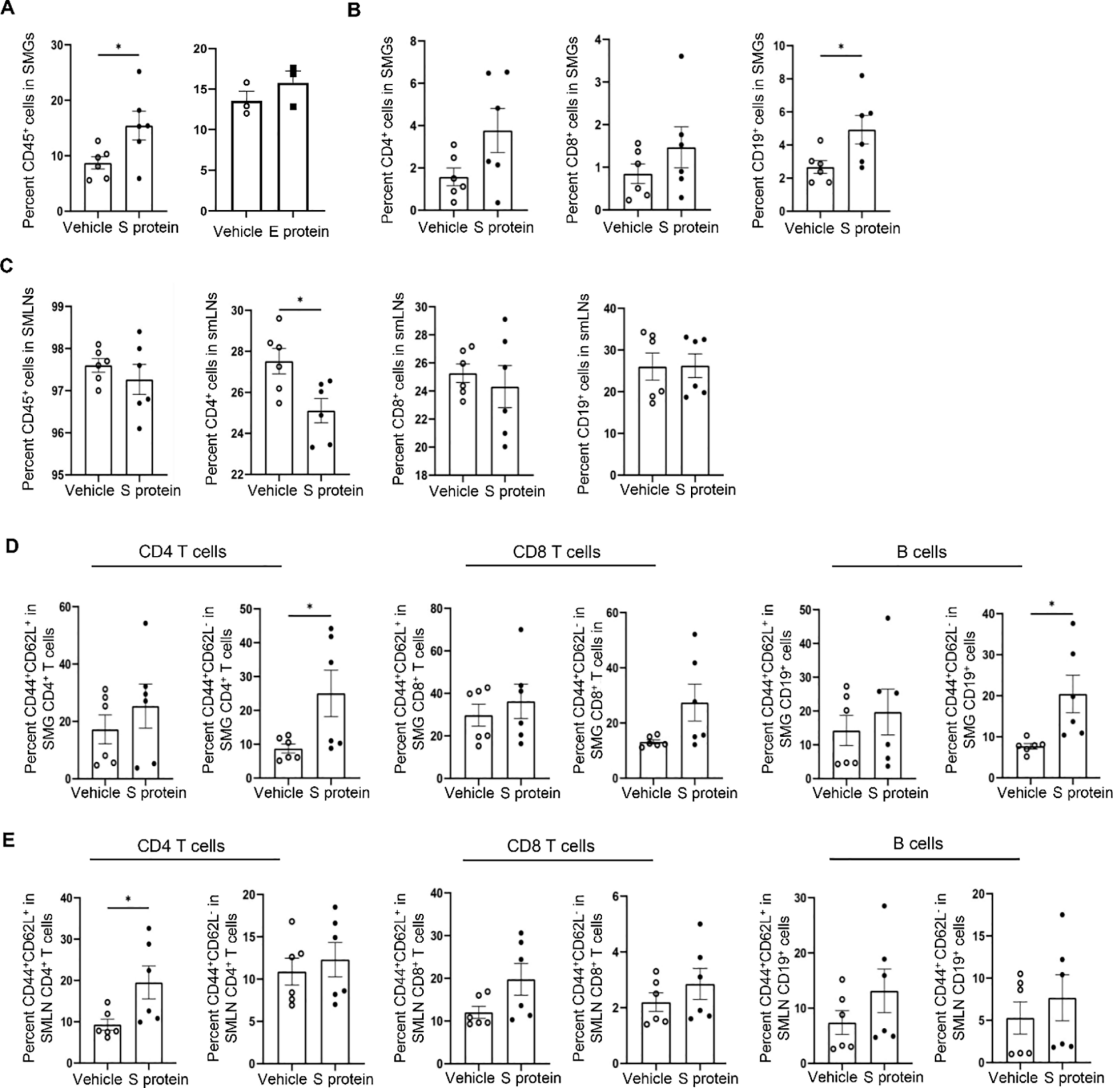
Intra-SMG administration of SARS-CoV-2 S protein increases the number of immune cells and activated T- and B cells within these glands. Mice were treated as described in **Figure 1**. **(A)** Percentages of total immune cells (CD45^+^) among live SMG cells. Left: Vehicle control versus S protein treatment. Right: Vehicle control versus E protein treatment. **(B)** CD4^+^ and CD8^+^ T cells, and B cells (CD19^+^) among SMG cells, and **(C)** those among cells from SMLNs as analyzed by flow cytometry (n=6 mice each group). **(D)** Percentages of effector/effector memory (CD44^high^CD62L^low^) and central memory (CD44^high^CD62L^high^) subsets within T and B cells within SMG cells and **(E)** those within cells from SMLNs, as determined by flow cytometry (n=6 mice/group). Data are presented as mean ± SEM. Statistical significance was determined using two-tailed Student’s t-test or Mann-Whitney U test as appropriate, with P values < 0.05 considered as significant. **P < 0.01.

To further characterize the immune alterations in SMGs and SMLNs induced by S protein and E protein treatments, we conducted flow cytometric analyses. The results indicated that S protein treatment significantly increased the percentage of total immune cells (CD45^+^) in SMGs compared with vehicle controls (**Figure 2A**, left), whereas E protein had no such effect (**Figure 2A**, right), consistent with the absence of changes in salivary flow rate following E protein treatment. In addition, S protein treatment significantly increased the percentage of B cells (CD19^+^) within SMGs, along with a non-significant trend toward higher frequencies of CD4 and CD8 T cells (**Figure 2B**). In SMLNs, the proportions of total immune cells, CD8 T cells, and B cells were comparable to those in the control group (**Figure 2C**), but there was a notable decrease in the proportion of CD4 T cells within SMLNs of the S protein-treated group (**Figure 2C**).

We simultaneously stained the cells for CD44 and CD62L (L-selectin) surface markers to distinguish the three major subsets of immune cells: naive (CD44^low^CD62L^high^), central memory (CD44^high^CD62L^high^), and effector memory (CD44^high^CD62L^low^) (48), which revealed alterations in the immune cells activities following S protein treatment. The effector memory (CD44^high^CD62L^low^) CD4 T cell subsets and the CD44^high^CD62L^low^ B cells subsets were significantly increased in SMGs following S protein treatment (**Figure 2D**). In addition, the proportion of the effector/memory (CD44^high^CD62L^low^) CD8 T cells in SMGs showed a non-significant trend of increase following S protein treatment (**Figure 2D**). There were no alterations in the effector/memory (CD44^high^CD62L^low^) subset of CD4 T, CD8 T or B cells with SMLNs, but there was a marked increase in the proportion of central memory (CD44^high^CD62L^high^) subset within CD4 T cells following S protein treatment compared to the control group (**Figure 2E**). Therefore, exposing SMG tissues to S protein leads to a significant increase in the activated T- and B cells in the glands.

### 3.3 Induction of salivary gland pathology by SARS-CoV-2 S protein is accompanied by increased phosphorylated STAT3 levels in the SMGs

**Figure 3:**
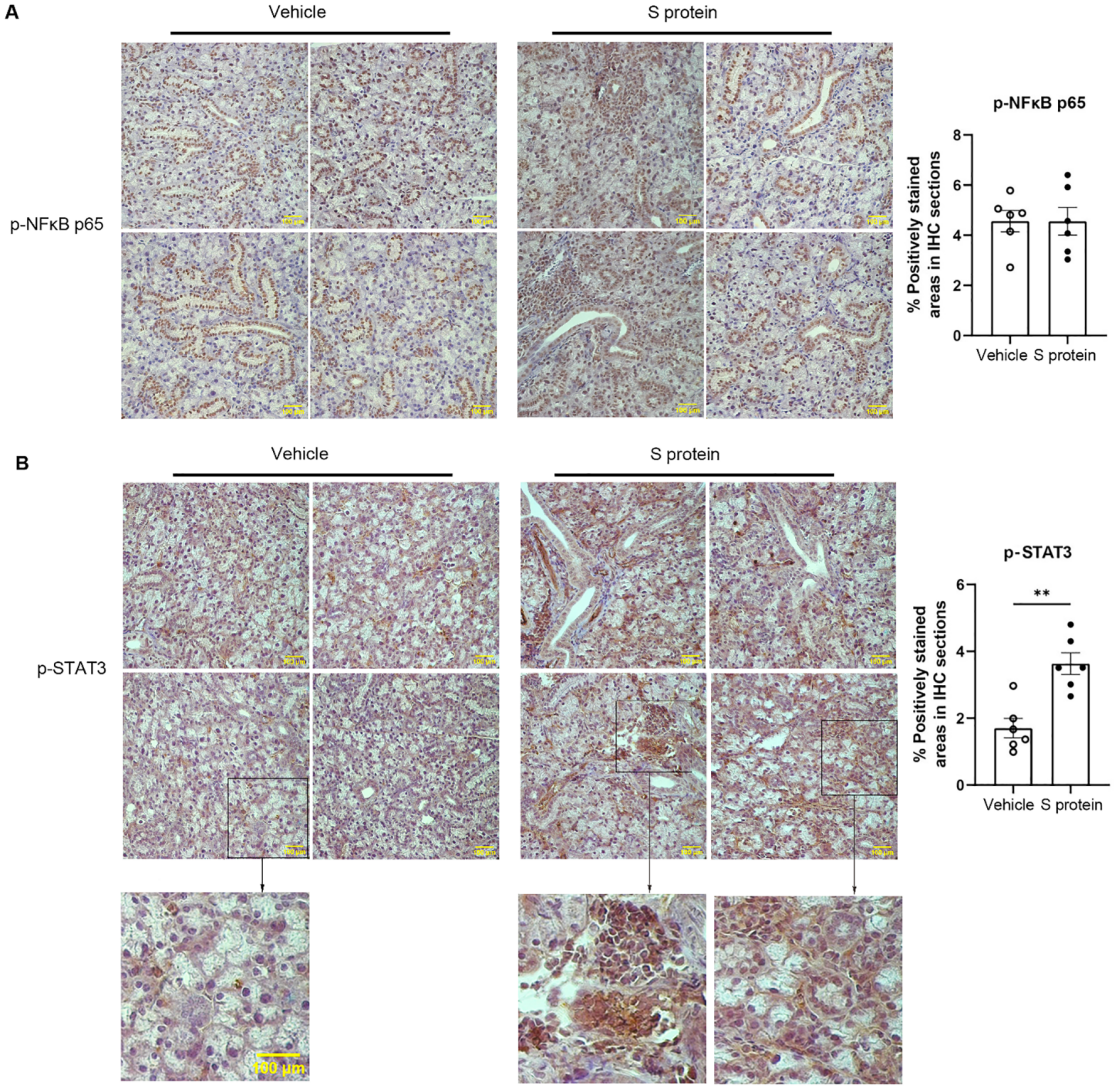
S protein-stimulation of salivary gland tissues increases the levels of phosphorylated STAT3 (p-STAT3) in the SMGs. Mice were treated as described in **Figure 1**. **(A)** Representative images of immunohistochemical (IHC) staining for phosphorylated NFκB p65 (p-NFκB p65) in SMG sections (X400 Magnification; n=6 mice each group). The graph shows the percentage of areas that are positively stained for p-NFκB p65. **(B)** Representative images of IHC staining for p-STAT3 in SMG sections (X400 magnification; n=6 mice/group), with magnified insets below showing selected regions from some of the images. Scale bars, 100 μm. The graph shows the percentage of areas that are positively stained for p-STAT3, quantified using the ImageJ software. Data are expressed as mean ± SEM. Statistical significance was determined using two-tailed Student’s t-test or Mann-Whitney U test as appropriate, with P values < 0.05 considered as significant. *P < 0.05, **P < 0.01.

To further elucidate the cellular and molecular mechanisms underlying S protein-triggered salivary gland pathology, we conducted immunohistochemical staining for TNFα, CXCL10, phosphorylated NFκB p65 (p-NFκB p65), phosphorylated STAT3 (p-STAT3), all of which are shown to be upregulated by S protein-treatment of lung tissues (23). Intriguingly, the levels of TNFα and CXCL10, two major mediators of immune responses and inflammation, were not significantly affected by S protein treatment (*p* = 0.157 and *p* = 0.818, respectively). Moreover, unlike previous findings in lung tissues, the levels of NFκB p65 in the SMGs remained unchanged following S protein exposure (**Figure 3A**). However, p-STAT3 levels in the SMGs were markedly upregulated following S protein treatments (**Figure 3B**), consistent with the reported effects of S protein on lung tissues (23). The p-STAT3 signals were primarily detected in infiltrating immune cells and cells surrounding acini, likely myoepithelial cells and salivary gland-resident macrophages (**Figure 3B**).

### 3.4 RNA-seq analysis reveals that S protein-induced salivary gland pathology is accompanied by marked molecular alterations in both epithelial and adaptive immune compartments.

**Figure 4:**
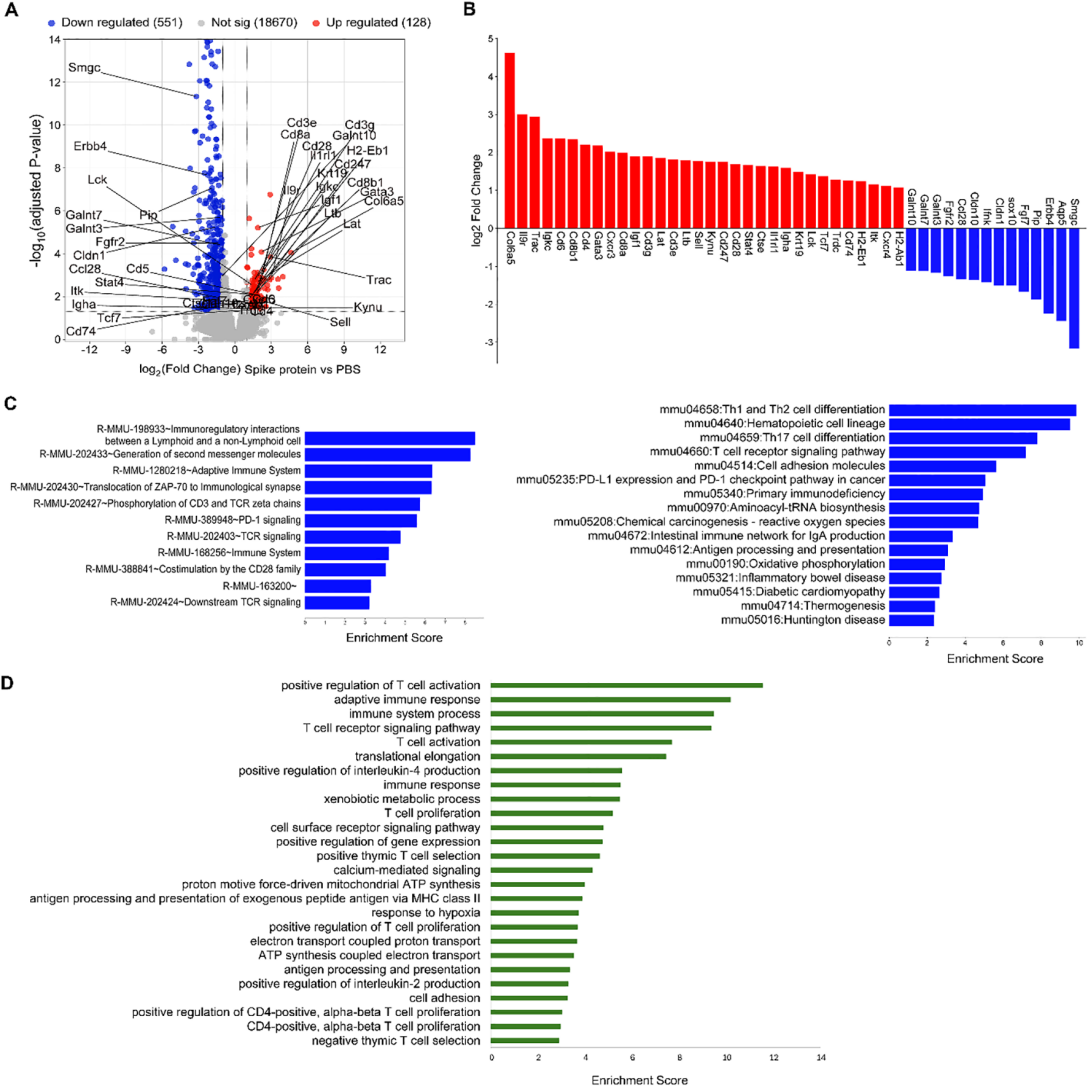
S protein-treatment significantly alters the transcriptomes of SMG cells. Mice were treated as described in **Figure 1**, and SMG cells were subjected to bulk RNA sequencing followed with bioinformatic analyses (n=3 mice/group). **(A)** Volcano plot showing all the identified differentially expressed genes (DEGs), with the upregulated and downregulated DEGs induced by S protein colored in red and blue, respectively. Selected DEGs of interest among those that are significantly changed (adjusted P value < 0.05 and absolute log_2_ fold change > 1) are labeled. **(B)** Bar graph shows selected DEGs of interest among those that are significantly changed (adjusted P value < 0.05 and absolute log_2_ fold change > 1). **(C)** Significantly enriched REACTOME pathways, left, and KEGG pathways, right, among the DEGs that were significantly upregulated by S protein (FDR < 0.05). **(D)** Significantly enriched biological processes, based on GO enrichment analysis, among the significantly upregulated DEGs by S protein treatment (FDR < 0.05).

To assess the molecular changes induced by S protein in salivary glands, we analyzed the differential gene expression in SMGs using bulk RNA sequencing. The Volcano plot (**Figure 4A**) illustrated the differentially expressed genes (DEGs) in the S protein-treated group compared to the vehicle-treated group. Selected DEGs pertinent to epithelial identity/function were labeled in the Volcano plot and also shown in the bar graph (**Figures 4B**). The results revealed a significant downregulation of key markers associated with epithelial identity, particularly acinar cells. These include *Aqp5, Pip, Sox10, Fgfr2*, and *Smgc*, which are critical for acinar cell identify/function; *Cldn1 and Cldn10*, encoding epithelial tight junction proteins; and *Galnt10*, encoding the polypeptide N-acetylgalactosaminyltransferase important for epithelial homeostasis and barrier function (**Figures 4A, B**). In parallel, RNA-seq data revealed significant alterations in adaptive immune responses in SMGs (**Figures 4A, B**). These include upregulation of genes involved in T and B cell signaling and activation, encompassing critical components of the TCR and BCR pathways, cytokines (e.g., *Ltb*), cytokine receptors (e.g., *Il9r, Il1rl1/St2*), chemokine receptors (e.g., *Cxcr3, Cxcr4*), and transcription factors (e.g., *Stat4, Gata3*) (**Figures 4A, B**). Moreover, there was a notable increase in genes involved in fibroblast activation and tissue fibrosis, such as *Col6a5* and *Igf1*. Taken together, these results strongly suggest that S protein treatment triggers an impairment of acinar cell function, disruption of epithelial barrier integrity, and enhancement of adaptive immune responses and tissue inflammation/remodeling within salivary glands.

We next performed multiple different pathway enrichment analyses, which strengthened the notion above and revealed additional pathways/processes enhanced by S protein treatment, such as innate immune responses and death, stress and remodeling of tissues. REACTOME Pathway analysis revealed that the most enriched pathways among S protein-upregulated genes include ‘Immunoregulatory interactions between a lymphoid and a non-lymphoid cell’, ‘Adaptive immune system’, and multiple T cell receptor components and signaling mediators (**Figure 4C**, left). KEGG pathway analysis indicated that the most enriched pathways among the S protein-upregulated genes include ‘Th1 and Th2 differentiation’, ‘Th17 differentiation’, ‘T cell receptor signaling pathway’, among others (**Figure 4C**, right). GO function and enrichment analysis showed that among the significantly upregulated DEGs, the most enriched biological processes (BP) include ‘Positive regulation of T cell activation’, ‘Adaptive immune response’, ‘T cell receptor signaling pathway’, ‘Positive regulation of interleukin-4 production’, among others (**Figure 4D**). Finally, GSEA analysis (**Supplementary Figure S1**) revealed that among the upregulated genes by S protein, the most enriched pathways include interferon-α/γ responses, IL-6–JAK–STAT3 and IL-2–STAT5 signaling, and complement/inflammatory pathways, suggesting enhanced adaptive and innate immune responses. Moreover, signatures linked to tissue stress and remodeling (apical junction, apoptosis, oxidative phosphorylation, ROS, hypoxia) were upregulated, suggesting death, stress and remodeling of salivary gland tissues.

Collectively, these findings highlight the dual impact of S protein on both the epithelial identity and the adaptive immune response within the salivary glands. The disruption of epithelial cell integrity, coupled with immune cell activation and tissue remodeling, underscores the complexity of S protein-induced salivary gland pathology and provides insights into the full spectrum of mechanisms by which SARS-CoV-2 induces the salivary gland disorder with autoimmune features.

## 4 Discussion

This study demonstrates that exposure of salivary glands to SARS-CoV-2 S protein induces glandular dysfunction and inflammation with SjD-like features in C57BL/6 mice, resembling those reported in COVID-19 patients (5, 8, 16, 49). Moreover, xerostomia is frequently reported in Long COVID patients (17, 18). S protein is key for viral entry into cells via ACE2, with proinflammatory properties (50) and readily detectable in salivary glands of post-COVID patients (5, 51). Our findings further establish the central role of S protein in SARS-CoV-2-induced pathology. Furthermore, the observation that S protein alone is sufficient to trigger salivary gland pathology with autoimmune features strongly suggests that persistent S protein presence and actions in salivary glands may critically contribute to the sustained salivary gland dysfunction and autoimmune-like features in Long COVID cases (17, 18).

Prior research on the role of S protein in COVID-19 mainly focused on its interaction with ACE2 (5, 50–52). A key novel finding from this study is that S protein can provoke salivary gland pathology independently of intact viruses and their interaction with ACE2, aligning with previous findings that S protein alone can trigger inflammation through cell surface TLR2/TLR4 in lung and brain tissues (23, 24, 45). Indeed, given that SARS-CoV-2 and its proteins cannot bind murine ACE2 (53), the salivary gland pathology induced by S protein observed here is conceivably independent of ACE2 and viral entry. Infections by viruses, such as EBV, CMV (4), HCV (3), and SARS-CoV-2 (5, 8), can trigger/exacerbate glandular inflammation and dysfunction. Previous mechanistic studies on the actions of these viruses primarily centered on the interaction of viral DNA/RNA with intracellular sensors such as RIG-I, MDA5, TLR3 and TLR7 (54). Our findings strongly suggest that S protein may also interact with cell surface sensors to drive the pathogenesis of salivary gland pathology following SARS-CoV-2 infection. It should be noted that even though E protein challenge did not alter salivary secretion and immune infiltration of salivary glands in this study, it may exert certain effects either alone or in combination with S protein, which warrants further investigations.

Both effector/effector memory CD4 T cells and B cells play key roles in viral immunity (55, 56) and chronic autoimmune inflammation, including SjD (38, 39, 57). It is therefore conceivable that the increase in these cells in salivary glands following S protein-challenge contributes to glandular inflammation and dysfunction. Future investigations should further elucidate the spatial relationships and crosstalk among various immune populations within leukocyte foci, and those between immune cells and salivary gland epithelial cells. Certain viruses are known to induce STAT3 signaling (58, 59). Studies using COVID-19 patient samples show that SARS-CoV-2-induced cytokine storm and pathology in various tissues are associated with activation of NFκB and JAK/STAT pathways, including STAT3 (60–64). We similarly observed an association between p-STAT3 and S protein’s effects in salivary glands, but p-NFκB p65 levels remain unchanged. Future studies using STAT3 inhibitors or genetic knockdown models would be valuable to confirm the role of STAT3 in mediating salivary gland alterations.

Viral infections are strongly associated with various autoimmune diseases (65) and exposure to EBV and CMV induces autoantibody production (6, 66). Importantly, the presence of ANA correlates with elevated S1‐specific antibodies in COVID‐19 patients during acute phase (46), and 20-30% of COVID-19 patients display serum ANA levels above the threshold typically associated with SjD (8). Consistent with these findings, our study showed that 33.3% of S protein-treated mice exhibited positive serum ANA coupled with increased amount of activated B cells in SMGs. These results suggest that S protein alone can induce notable B cell production of ANA, aligning with the uptick in SjD cases following the COVID-19 outbreak (65, 67).

Our transcriptomic analyses reveal the impact of S protein on both epithelial and immune compartments of salivary glands, with a downregulation of acinar epithelial markers and tight junction genes, and upregulation of pathways associated with tissue apoptosis, stress and remodeling, including fibrosis and adipogenesis. In addition, there was robust upregulation of genes/pathways characteristics of T and B cell immune responses, TCR and BCR signaling, cytokines and chemokine receptors, Th1/Th2/Th17 differentiation, IFN responses, and chronic inflammation. While effector T cell responses are consistently demonstrated by both flow cytometry and transcriptomics, multiple genes/pathways also converge on B cell activation and autoantibody production, including upregulation of *Ltb* (lymphotoxin-β), a key organizer of ectopic lymphoid structures, IL-6–JAK–STAT3 signaling, and Th2 response. They provide a potential mechanistic basis for S protein-induced emergence of ANA. Together, these results highlight the impact of S protein that impairs salivary epithelial identity/function while driving adaptive autoimmune responses and tissue remodeling, and offer mechanistic insights into how SARS-CoV-2 induces autoimmune-like salivary gland disorder.

A key question for future research is to identify the primary cell types that S protein directly interacts with to trigger the salivary gland pathology we observed. Our findings strongly suggest that salivary gland epithelial cells may be primary responders to S protein and with their perturbation subsequently promotes activation and/or chemoattraction of immune cells. RNA-seq analysis reveals that, in addition to heightened adaptive immune responses, S protein triggering causes a profound downregulation of gene signatures of acinar cells, such as *Aqp5*, *Smgc*, *Pip* and *Sox10* (19, 68–72). Previous studies also indicated that S protein alone can induce inflammatory responses in macrophages, lung epithelial cells, and microglial cells through TLR2/TLR4 (23–25). Notably, salivary gland epithelial cells in healthy humans express TLR2 and -4 proteins (19, 26, 27), which are upregulated in SjD (19, 26, 27, 73). TLR2 and TLR4 mRNAs are detected across multiple ductal and acinar subsets in healthy humans (74). We postulate that epithelial cells may be the primary targets of S protein in salivary glands, and that S protein may interact with TLR2/TLR4 on these cells to impair their function and enhance their immune-activating properties. Future functional studies using genetic or pharmacologic ablation of TLR2/TLR4 will help delineate the contribution of these pathways to S protein-induced salivary gland pathology and epithelial-immune crosstalk. The knowledge obtained may guide the development of treatment and management strategies for post-COVID-associated salivary gland disease by targeting S protein, its interaction with epithelial cells (e.g., S protein-TLR2/4 axis), and downstream inflammatory cascades.

There are several additional limitations in this study. Although we showed that intra-SMG injection of S protein can trigger glandular inflammation and dysfunction, this focused, reductionist model does not recapitulate the full pathophysiological context of SARS-CoV-2 infection. Future studies using virus infection models combined with ablation of candidate pathways will be needed to define the translational relevance of these findings. This study only assessed the effects of S protein at a single time point, and analyses over longer treatment durations with more time points will further clarify the actions of S protein during both acute and chronic phases post-infection. Another limitation is that beyond ANA, other autoantibodies, such as SSA/Ro and SSB/La, were not assessed. A more comprehensive evaluation of SjD-associated autoantibodies will provide deeper insights into the autoimmune B cell responses in this setting. Finally, we only used female mice in this investigation due to the striking female predominance of SjD as well as higher ANA levels in female COVID-19 patients compared to the male counterparts (8, 20, 37, 39, 40). Future studies in male subjects will be important to fully elucidate potential sex-dependent effects of S protein on salivary glands.

In conclusion, this study shows, for the first time, that SARS-CoV-2 S protein alone is sufficient to initiate considerable pathology in salivary glands of C57BL/6 mice, with autoimmune features resembling SjD and observed in COVID-19 and Long COVID patients. Moreover, we identified key cellular and molecular alterations that may contribute to S protein-mediated effects. These findings provide new insights into the roles of SARS-CoV-2 S protein in chronic salivary gland autoimmune inflammation.

## Data Availability

The RNA-seq data generated in this study have been deposited in the NCBI Sequence Read Archive under BioProject accession PRJNA1358258.
