# Supplementary material for: SARS-CoV-2 spike protein induces salivary gland dysfunction and immune infiltration in C57BL/6 mice
Source: Front Immunol. 2025 Nov 21;16:1667597. doi: 10.3389/fimmu.2025.1667597 (PMC12678246; doi:10.3389/fimmu.2025.1667597)
Supplement: Supplementary file 1 [file DataSheet1.pdf]

Supplementary Materials

Manuscript title: SARS-CoV-2 Spike Protein Induces Salivary Gland Dysfunction and Immune Infiltration in C57BL/6 Mice

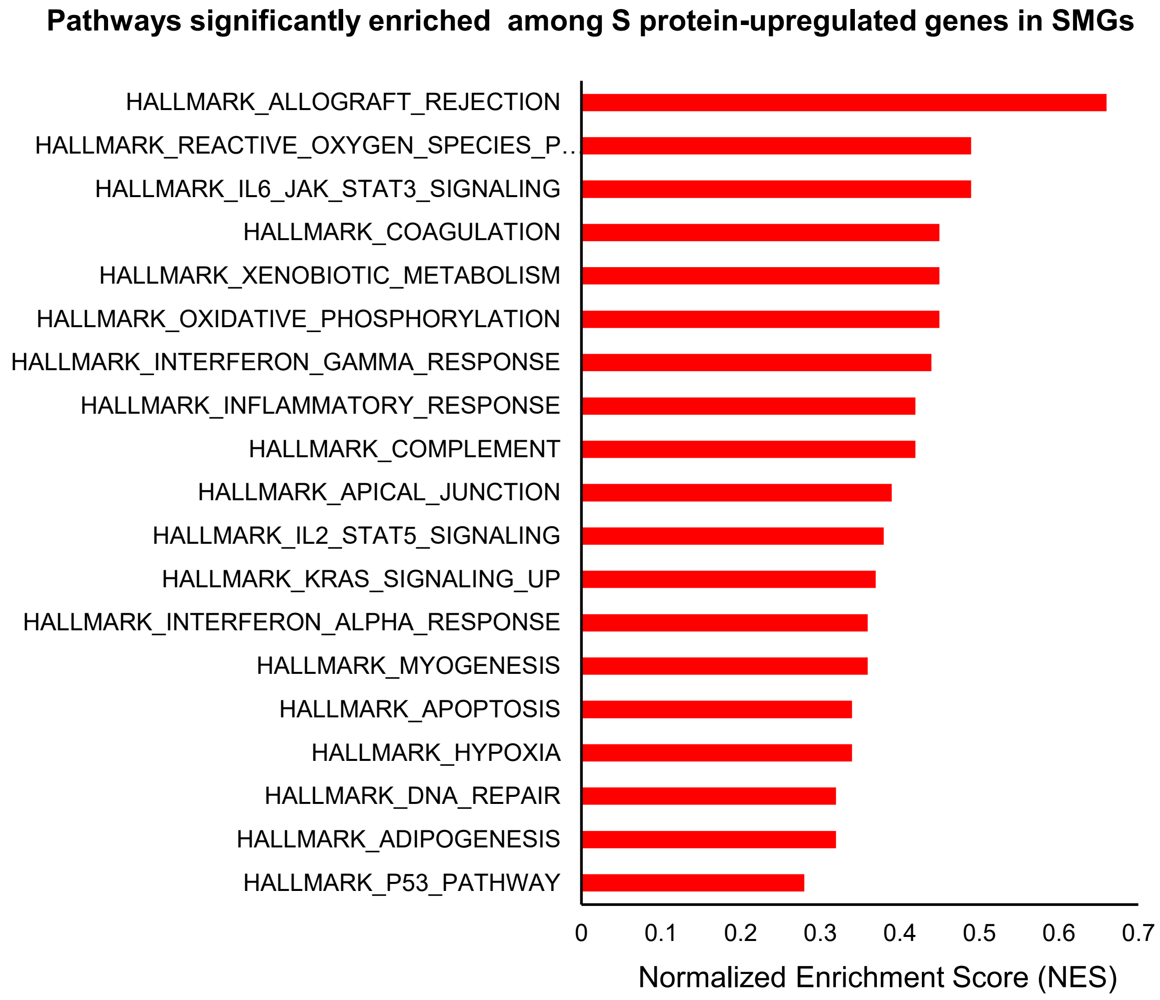

Supplementary Figure 1. Gene Set Enrichment Analysis (GSEA) showing significantly enriched pathways among upregulated genes in SMG cells following S protein treatment. GSEA was performed on RNA sequencing data described in Figure 4 using the Molecular Signatures Database (MSigDB) Hallmark gene set collection (mouse, h.all.v25.1.hs.symbols.gmt). All the pathways shown are significantly enriched (FDR $q < 0.05$) among DEGs upregulated by S protein in SMGs and presented based on normalized enrichment scores (NES).
